# Fragment-Based Discovery of Inhibitors of the Bacterial DnaG-SSB Interaction

**DOI:** 10.3390/antibiotics7010014

**Published:** 2018-02-22

**Authors:** Zorik Chilingaryan, Stephen J. Headey, Allen T. Y. Lo, Zhi-Qiang Xu, Gottfried Otting, Nicholas E. Dixon, Martin J. Scanlon, Aaron J. Oakley

**Affiliations:** 1 Molecular Horizons and School of Chemistry, University of Wollongong, and Illawarra Health and Medical Research Institute, Wollongong, NSW 2522, Australia; zorik@uow.edu.au (Z.C.); tyl667@uowmail.edu.au (A.T.Y.L.); zhiqiang@uow.edu.au (Z.-Q.X); nickd@uow.edu.au (N.E.D.); 2 Monash Institute of Pharmaceutical Sciences, Monash University, Parkville, VIC 3052, Australia; stephen.headey@monash.edu (S.J.H.); martin.scanlon@monash.edu (M.J.S.); 3 Research School of Chemistry, Australian National University, Canberra, ACT 2601, Australia; gottfried.otting@anu.edu.au

**Keywords:** antibacterial agents, fragment-based screening, primase, protein–protein interactions, SSB

## Abstract

In bacteria, the DnaG primase is responsible for synthesis of short RNA primers used to initiate chain extension by replicative DNA polymerase(s) during chromosomal replication. Among the proteins with which *Escherichia coli* DnaG interacts is the single-stranded DNA-binding protein, SSB. The C-terminal hexapeptide motif of SSB (DDDIPF; SSB-Ct) is highly conserved and is known to engage in essential interactions with many proteins in nucleic acid metabolism, including primase. Here, fragment-based screening by saturation-transfer difference nuclear magnetic resonance (STD-NMR) and surface plasmon resonance assays identified inhibitors of the primase/SSB-Ct interaction. Hits were shown to bind to the SSB-Ct-binding site using ^15^N–^1^H HSQC spectra. STD-NMR was used to demonstrate binding of one hit to other SSB-Ct binding partners, confirming the possibility of simultaneous inhibition of multiple protein/SSB interactions. The fragment molecules represent promising scaffolds on which to build to discover new antibacterial compounds.

## 1. Introduction

Duplication of chromosomal DNA prior to cell division is a fundamental process in living cells. During initiation of DNA replication in *Escherichia coli*, DnaB helicase is loaded with the assistance of the helicase loader DnaC onto double-stranded DNA and unwinds it [1,2]. DnaB, through direct physical interaction with DnaG primase, forms the primosome [3], which uses its primase activity to synthesize short RNA primers essential for the function of DNA polymerase III [4,5]. 

DnaG is a DNA-dependent RNA polymerase [6]. In bacteria it is comprised of three distinct domains: an N-terminal zinc-binding domain (ZBD) responsible for DNA template recognition [7], a central catalytic domain (RNA polymerase domain, RPD) [8,9], and a C-terminal helicase-binding domain (HBD or DnaGC), which is responsible for interaction with DnaB helicase and single-stranded DNA-binding protein (SSB) [10,11,12]. The crystal and solution structures of DnaGC of *E. coli* were determined by X-ray crystallography as a non-physiological domain-swapped dimer [13] and as a monomer in solution by NMR spectroscopy [14]. 

SSB protects single-stranded DNA during DNA replication. It is an interaction hub known to bind to more than 14 other proteins involved in various stages of DNA replication, repair, and recombination through a highly conserved C-terminal hexapeptide motif (SSB-Ct, sequence: DDDIPF) [15,16]. SSB’s binding partners include DnaG [10,12], the Pol lll χ subunit [12,17,18,19,20,21,22], the PriA replication restart helicase [23], and exonuclease I [24,25].

The SSB-Ct binding site in DnaGC has been identified by NMR. The binding pocket is formed by basic residues K447, R452, and K518, as well as T450, M451, I455, and L519 [26]. Moreover, the DnaGC point mutants K447A, T450A, R452A, and K518A dramatically attenuate SSB-Ct binding. Mutagenesis and NMR experiments, in particular ^15^N–^1^H heteronuclear single-quantum correlation (^15^N–^1^H HSQC) experiments suggested that the conserved R452 residue forms a salt bridge with the carboxylic acid of the C-terminal Phe residue of the SSB-Ct, whereas the other positively charged residues around the binding pocket interact with the negatively charged residues of SSB-Ct. The SSB-Ct binding pockets in other SSB-binding proteins have characteristics in common with the binding pocket in DnaGC; e.g., those in ExoI [24], RecO [27], Pol lll χ [22], and PriA [23]. 

The SSB-Ct binding pockets in some or all of these proteins have been suggested to be very good targets for development of new antibacterial agents because many of the interactions are essential for bacterial survival and resistance to compounds that interfere with multiple interactions could not easily develop by target mutagenesis [16]. This argument depends critically on a single compound mimicking the SSB-Ct peptide sufficiently well that it is able to bind tightly to three or more essential binding pockets that are lined with different residues and thus have structures that are more or less distinct. The observed gross structural similarities among pockets in SSB-Ct binding partners, including the ionic interaction with the C-terminal Phe and the basic rim that interacts with the acidic residues suggest such compounds might exist, but the only useful way to establish this for sure is to quantify the binding to multiple potential targets of compounds selected against one of them. 

To begin the process of determining whether SSB-Ct binding pockets are actually suitable targets, we report the use of fragment-based screening (FBS) to find compounds binding to *E. coli* DnaGC. FBS uses small (<300 Da) compounds called “fragments” as starting points for drug discovery. Several biophysical methods may be used in fragment screening [28]. Here, we report the use of surface plasmon resonance (SPR) and NMR measurements to screen for binders that target the SSB-binding pocket in DnaGC.

## 2. Results and Discussion

### 2.1. Screening of Fragment Libraries

An SPR competition assay (example in Figure S1a) was used as the first-pass screen. It identified six small-molecule fragments that competed with immobilized SSB-Ct peptide for binding to a N-terminally truncated DnaG protein missing just the ZBD; we call this protein DnaG-RCD, comprised of the RPD and DnaGC domains. These fragments were validated by saturation-transfer difference (STD) NMR [29], where transient binding of the ligand to the protein is detected by attenuation of the ligand NMR spectrum (example in Figure S1b). In parallel with the confirmation of SPR hits, cocktails encompassing the complete MIPS library of 1140 structurally diverse fragments [30] were independently screened by STD-NMR. Fragments identified in cocktails were re-tested as pure ligands in the STD-NMR assay. The hits identified were ranked according to STD signal intensity. In total, 56 fragments were identified as top hits (rank 3, clear hit, strong intensity difference) and 62 as rank 2 (clear hit, moderate intensity) [30]. From the final STD rank 3 and 2 compounds together (80 compounds), about 50 fragments were identified as “frequent hits” or “PAINS” [30] and were excluded from further screening.

### 2.2. Validation of Fragment Binding by 2D NMR

**Figure 1:**
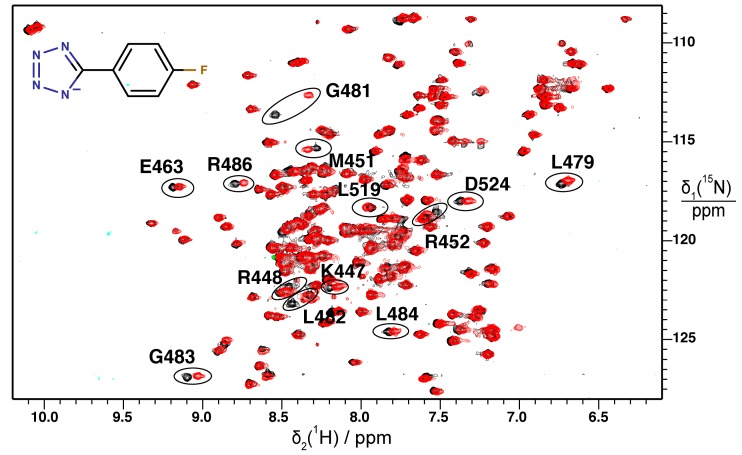
Superimposition of ^15^N–^1^H HSQC spectra of DnaGC. The protein spectrum in the absence of fragment in black is compared with its spectrum after addition of fragment **4** (structure shown) in red. Representative assignments of resonances that showed the highest weighted chemical shift perturbation (CSP) (Figure S3d) are shown.

Two-dimensional (2D) ^15^N–^1^H HSQC spectra of the uniformly ^15^N-labeled DnaGC domain were employed to validate STD hits. Buffer conditions were optimized, and no major differences were observed in recorded spectra of the protein alone compared to the original studies [13,14]. We were able to assign some of the missing resonances for residues forming the SSB-Ct binding pocket in the spectrum of the apo-protein reported by Naue et al. [26]; i.e., those of K447, R448, T449, N511, and N565. ^15^N–^1^H HSQC spectra were recorded on 0.1 mM solutions of ^15^N-DnaGC mixed with compounds at 3.3 mM. The compounds were ranked according to the magnitude of the generated weighted chemical shift perturbation (CSP; Section 3.5), and the best four were selected (Figure 1, Figures S2a–c and S3a–d).

**Figure 2:**
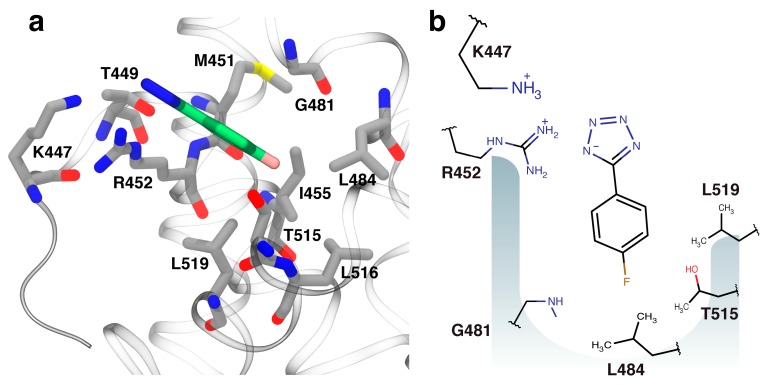
Modeled orientation of fragment **4**. (**a**) The docked orientation of fragment **4** (green carbon atoms) in the single-stranded DNA-binding (SSB)-Ct binding pocket of DnaGC (gray carbon atoms). (**b**) A schematic representation of interactions between fragment **4** and its binding pocket. In all structural figures, the protein was visualized using visual molecular dynamics (VMD) [31].

By monitoring the protein chemical shift and peak intensity changes upon addition of compound and mapping the CSP onto the protein surface, the location of the ligand-binding pocket could in each case be identified as the SSB-Ct binding pocket of DnaGC (Figure 2 and Figure S4).

**Figure 3:**
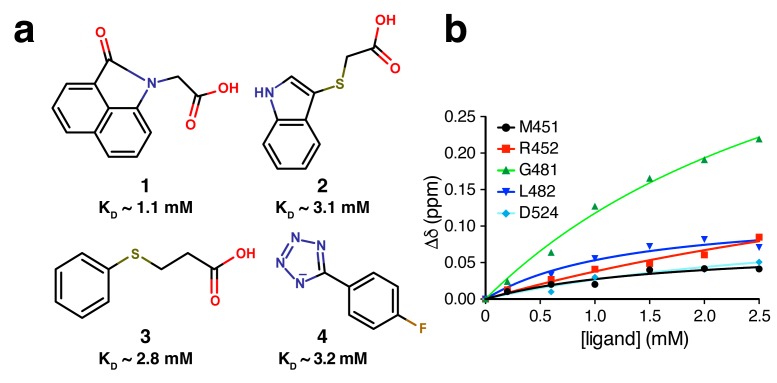
(**a**) Structure of hits with binding affinities for further optimization. (**b**) ^15^N–^1^H HSQC titration of fragment **4**. Binding affinities (*K*_D_ values) were derived from the change in chemical shift, Δδ, with incremental additions of ligand.

Comparison of the four hits revealed some similar features such as the presence of indole groups in fragments **1** and **2** or an aliphatic thioether-linked extension in **2** and **3**. In addition, three (**1**–**3**) have a carboxylate attached (Figure 3a) and fragment **4** has a 1*H*-tetrazole group, which is a carboxylate bioisostere [32]. Tetrazoles have p*K*_a_ values comparable to carboxylic acids (~5) and are good hydrogen bond acceptors. Modeling studies suggest that deprotonated tetrazoles form stronger hydrogen bonds than carboxylate groups [33].

Affinity measurements of weakly binding ligands are challenging for most biophysical techniques [34,35]. Nevertheless, binding affinities of fragments **1**–**4** can be roughly estimated to be in the 1–3 mM range by NMR titration experiments that monitored the gradual change in chemical shift of a few well-resolved resonances in HSQC spectra (Figure 3b).

### 2.3. Orientation of Identified Hits Using Molecular Docking

The docked structure of fragment **4** bound into DnaGC (Figure 2a) is in agreement with the chemical shift perturbation (CSP) data. The tetrazole anion of **4** makes hydrogen bonds and/or a salt bridge with the side chains of K447, T449, and R452. The phenyl ring appears likely to mimic the last phenylalanine residue in the SSB-Ct peptide and makes hydrophobic interactions with P480, G481, T515 and L519 in the binding site. Fluorine is a strong hydrogen-bond acceptor [36], and the model suggests that there is a hydrogen bond to the *para*-fluoro group from the amide proton of G481. Methyl groups from the side chains of L448, L455, T515, L516, and L519 also interact with the *para*-fluoro group.

In the case of fragment **1**, CSP-guided docking predicts that the carbonyl group interacts with the side-chain of R452. The fragment orientation enables favorable contact of the free carboxylate of the fragment and the positively charged side chain of K518 (Figure S4). Its improved binding affinity (~1.1 mM) is most likely explained by its hydrophobic skeleton occupying the shallow binding pocket. Comparison of fragment 1 with an analog where the carboxylate is substituted with a methyl group gave fewer peak shifts in the HSQC spectrum, confirming that the carboxylic acid group improves the binding (Figure S5). 

Fragments **2** and **3** have similar aliphatic chains with thioether links to aromatic groups. As with other fragments, the carboxylate moieties are predicted to form electrostatic and hydrogen bond interaction, whereas the main chemical backbone mimics the phenylalanine residue in SSB-Ct. Thioethers may have a strong influence on the conformation of aliphatic chains [37]. In the proposed binding mode of fragment **3**, the sulfur atom interacts with R452 while the carboxylate forms hydrogen bonds with I455 and G481 (Figure S6).

One of the fragments tested, *N*-acetylated L-Phe, is expected to mimic the C-terminal residue (F177) in the SSB-Ct peptide. STD and ^15^N–^1^H HSQC experiments confirmed the binding event (data not shown). However, binding affinities measured in titration experiments monitored by HSQC spectra showed that it has about two-fold lower affinity compared to other hits (**3**, **4**). The modeled orientations of the fragment show indeed that the phenyl ring forms hydrophobic contacts in the binding site, and the carboxylate interacts with R452 (Figure S7). Substituting the carboxylate with a 1*H*-tetrazole ring, as found in fragment **4**, increased the affinity 2-fold.

### 2.4. Fragment-to-Hit Optimization

**Figure 4:**
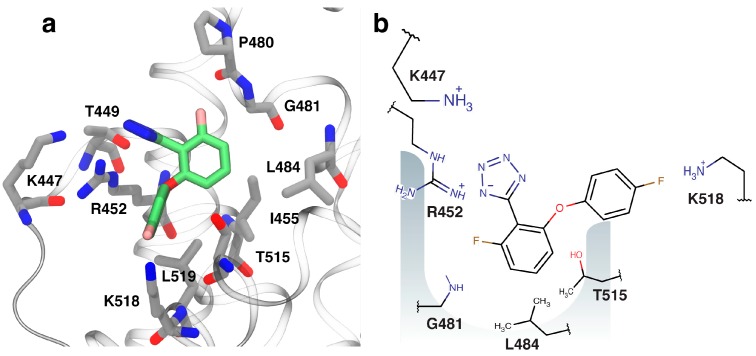
Visualization of binding of compound **5**. (**a**) The lowest energy binding poses of **5** (green carbon atoms) bound to DnaGC (gray carbon atoms). (**b**) Schematic representation of residues involved in interaction with compound **5**.

With the knowledge that the tetrazole moiety might confer improved membrane-crossing properties compared with carboxylates owing to its higher lipophilicity, fragment **4** (Figure 3a) was chosen as a starting point for fragment-to-hit optimization. As docked into DnaGC, **4** has suitable vectors for fragment growth. The in silico analog screen identified 10 tetrazole analogs with favorable binding poses relative to the SSB-Ct peptide (not shown). Nevertheless, STD and 2D NMR experiments showed that most of these analogs did not bind to DnaGC. As an exception, 5-[2-fluoro-6-(4-fluorphenoxy)phenyl]tetrazol-1-ide (**5**) showed a STD signal and significant CSP in 2D NMR experiments (Figure S3e). Mapping of the CSP on the protein surface allowed a binding pose of compound **5** to be calculated (Figure 4).

**Figure 5:**
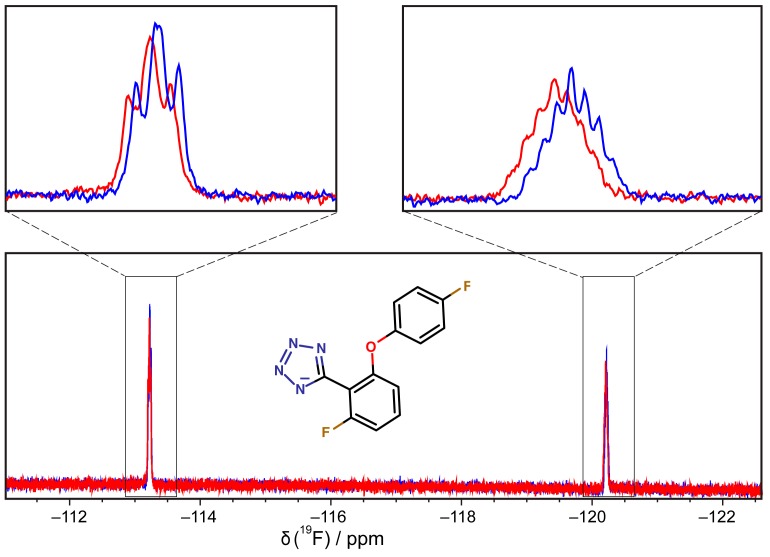
1D ^19^F-NMR spectra of compound **5** at 1 mM in the presence (red trace) and absence (blue trace) of 50 μM DnaGC.

The observed NMR peak shifts were consistent with the docked orientation. The negatively charged tetrazole is predicted to form favorable electrostatic and hydrogen bond interactions with the side chains of K447 and R452. The methyl groups of L446, M451, L455, L484 T515 and L519 form a hydrophobic pocket accommodating two aromatic rings of the compound. One of the fluorine atoms is about 3 Å from the amide group of G481 while the second at the *para*-position of the phenoxy group points out of the pocket toward K518. In the docked conformation, these fluorine atoms are involved in hydrogen bond formation with these two residues (Figure 4). To test the docked orientation of **5**, 1D ^19^F-NMR was carried out using a 20-fold excess of compound over protein. Fluorine signals were broadened and shifted slightly downfield, confirming binding of **5** to DnaGC (Figure 5). Nevertheless, the 3D NOESY-^15^N-HSQC spectrum failed to detect any protein–ligand NOEs.

**Figure 6:**
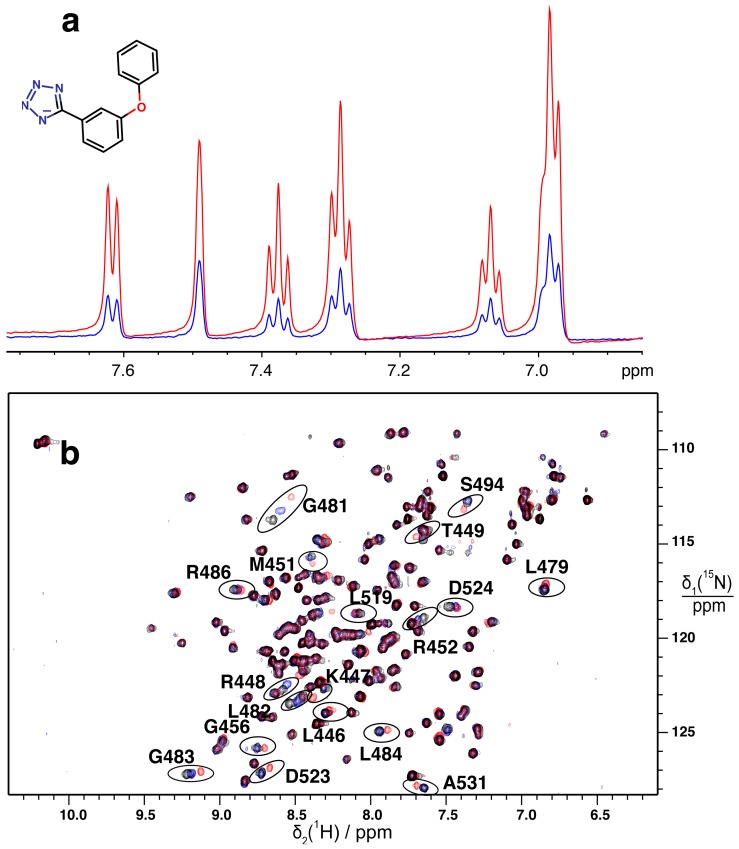
(**a**) Saturation transfer difference (STD) spectrum of compound **6** using DnaG-RCD. In red is a 1D ^1^H-NMR reference spectrum, overlaid with a STD spectrum (blue). (**b**) Overlay of ^15^N–^1^H HSQC spectra of ^15^N-DnaGC (black) with **5** (blue) and **6** (red), each at 1 mM. The apo-protein spectrum is shown in black. Representative assignments of resonances that showed the highest weighted CSP (Figure S3e,f) are shown.

Compound **5** was shown to bind to DnaGC with about a three-fold improved affinity compared to the starting fragment **4**, as measured by titration experiments monitored by ^15^N–^1^H HSQC spectra (*K*_D_ = 1.3 mM). Searching the *ZINC* database [38] for ligands structurally similar to **5** indicated that the only available analog was **6**. It is missing the halogen atoms and has a *meta*-phenoxy group (Figure 6). The binding of **6** to DnaGC was also assessed by STD and ^15^N–^1^H HSQC experiments (Figure 6).

Compounds **5** and **6** showed similar patterns of CSPs (Figure S3e,f). Compounds that possess similar protein-binding modes are known to induce similar CSPs [39]. Changes in the position of the phenoxy group in **6** relative to **5** increased the magnitude of the CSP as a result of a slightly improved binding affinity, *K*_D_ = 1.2 mM.

**Figure 7:**
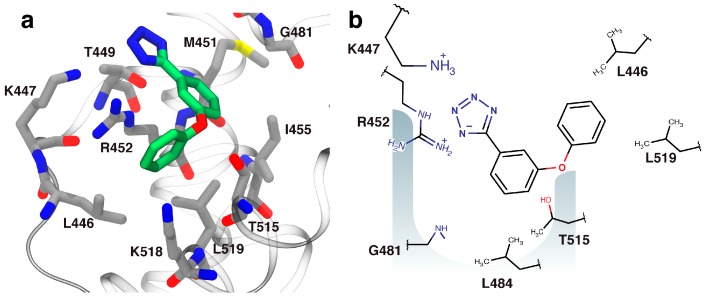
(**a**) Docked binding pose of **6** (green carbon atoms) bound to DnaGC (gray carbon atoms). (**b**) Schematic representation of interactions.

Compound **6** was in turn docked to DnaGC and is predicted to form electrostatic interactions with the side chains of K447, T449 ad R452 (Figure 7). The central aromatic ring sits in a hydrophobic groove formed by M451, I455 and L484, while the phenoxy oxygen atom forms a hydrogen bond with the side chain –OH of T515. In addition, the phenoxy ring forms a cation–π interaction with the guanidinium group of R452 and hydrophobic contacts with the L446 and W522 side chains. 

**Figure 8:**
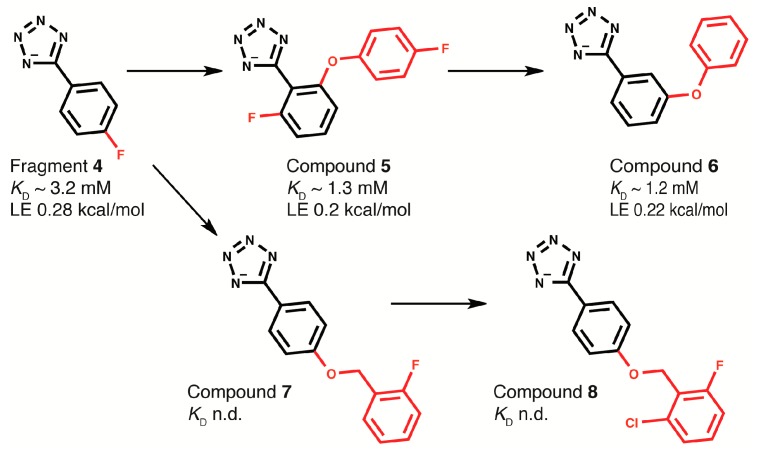
Schematic representation of optimization of fragment **4**. The red labeled groups were added during fragment-to-hit optimization. LE: Ligand efficiency (∆*G*/[number of heavy atoms]), n.d.: not determined.

To sample the impact of substituents at the *para*-position of the phenyl group of **4**, additional compounds were purchased (compounds **7** and **8**; Figure 8). Molecular docking suggested that *para*-substitutions might dramatically change the orientation of the tetrazole moiety in the binding pocket, and STD and ^15^N–^1^H HSQC experiments showed that replacing the *para*-fluorine with a bulky substituent reduced the CSPs substantially.

### 2.5. Binding of Compounds to Other SSB Partner Proteins

**Figure 9:**
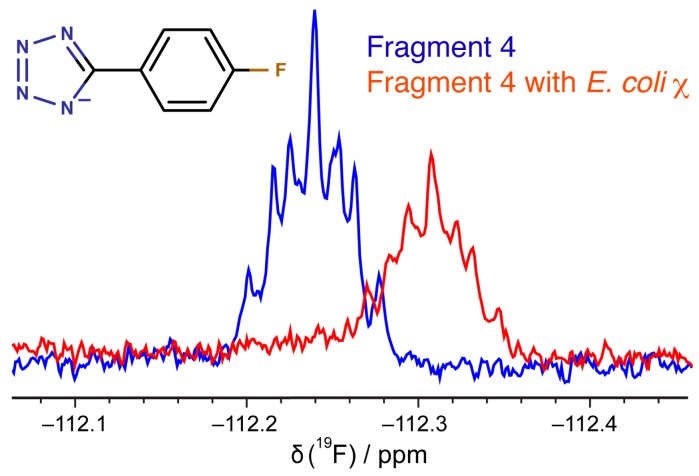
1D ^19^F-NMR spectra. The blue spectrum is of fragment **4** alone and its spectrum in the presence of *E. coli* χ is shown in red.

The fragments identified here and the first generation of optimized leads for DnaGC were tested against other SSB-Ct binding partners including *E. coli* PriA, *E. coli* RNAse HI, and the χ subunit of *E. coli* and *Acinetobacter baumannii* DNA polymerase III. STD-NMR was used to assess binding. All of the identified fragments showed STD signals, confirming binding (Figure S8). Moreover, the docked orientation of fragment **4** in the binding site of *E. coli* χ showed the possibility of hydrogen bonding with the fluorine atom. 1D ^19^F NMR (Figure 9) demonstrated binding.

## 3. Materials and Methods 

### 3.1. Protein Expression and Purification

A phage λ-promoter plasmid (pZX1404) that directs overexpression of a protein comprising the central and C-terminal domains of *E. coli* DnaG primase (residues 111–581, here called DnaG-RCD) was constructed by cloning a PCR fragment between the *Bam*HI and *Eco*RI sites of vector pND706 [40]. PCR was performed using plasmid pPL195 [41] as template and the following primers (restriction sites in italics): dnaG_RCD_F, 5′-GCGGGATCCTAAGAAGGAGATATA*CATATG* ACGCTTTATCAGTTGATG; dnaG_RCD_R, 5′-GCG*GAATTC*TTACTTTTTCGCCAGCTC C. The full sequence of the gene encoding RCD was then verified by nucleotide sequence determination. Another plasmid pZX1399 encoding amino acids 115–581 of *E. coli* DnaG was also constructed in a similar manner. However, the protein was expressed in insoluble form, and therefore was not used. Unlabeled DnaG-RCD and unlabeled and ^15^N-labeled DnaGC were expressed and purified as described previously for DnaGC [42].

### 3.2. Fragment Libraries

The “first pass screen” fragment library (Zenobia Therapeutics, San Diego, CA, USA) was used for the SPR competition assay. Each fragment (50 mM in DMSO) was diluted to 1 mM final concentration. Fragment library members were tested for chip surface binding to eliminate false positives. 

The Monash Institute of Pharmaceutical Science (MIPS) library comprised of around 1140 fragments purchased from Maybridge was used for STD-NMR experiments. The individual fragments were diluted in ^2^H_6_-DMSO to give ~660 mM final stock concentrations [30]. The fragments were mixed in cocktails of up to 6 compounds with well-resolved resonances in their 1D ^1^H-NMR spectra.

### 3.3. SPR Competition Assay

SPR measurements utilized a Biacore T200 instrument (GE Healthcare, Little Chalfont, UK) at 20 °C to measure the competition of compounds for the DnaGC/SSB-Ct peptide interaction. The buffer contained 10 mM HEPES (pH 7.4), 3 mM EDTA, 100 mM NaCl, 2% DMSO, 1 mM dithiothreitol and 0.05% (*v*/*v*) surfactant P20 (GE Healthcare). An N-terminally biotinylated SSB-Ct peptide [Biotin-(Ahx)-GSAPS-NEPPMDFDDDIPF; where Ahx is an amino-hexanoate spacer, followed by the 16 C-terminal residues of SSB highlighted in bold] was immobilized onto a streptavidin (SA) chip surface. RCD at 30 μM and fragments at 1 mM concentrations were used in all SPR experiments. Each sample was mixed for approximately 15 min prior to measurements. Mixtures were injected separately onto two flow cells, one of which served as a reference. 

Prior to measurements, each individual fragment was tested for solubility and non-specific binding to an unmodified surface at 1 mM concentration to eliminate false positive responses. Compounds that bound non-specifically to the chip surface were excluded from screening. A flow rate of 5 μL/min was used during the 60 s injection and 60 s dissociation phases for all experiments. 

### 3.4. Saturation-Transfer Difference (STD) NMR Spectroscopy

STD-NMR experiments were carried out using 5 μM unlabeled DnaG-RCD and mixtures of 6 fragments in each sample, at ~250 μM for each fragment. The sample volume was 500 μL with 98–99% ^2^H_2_O buffer containing 50 mM phosphate (pH 7.8), 50 mM NaCl and 1 mM dithiothreitol. Spectra were recorded at 283 K using a Bruker Avance 600 MHz spectrometer (Bruker, Karlsruhe, Germany) equipped with a cryoprobe. Saturation of protein was achieved with a 4 s Gaussian pulse sequence train centered at −1 ppm. For reference spectra, a similar saturation pulse was applied 20 kHz off-resonance. A 20 ms spin-lock period was applied before acquisition to allow the residual protein signals to decay. The STD experiments were recorded over 64 scans. All NMR data were processed using TOPSPIN 3.1. Relative intensities were based on the most intense STD signal (I_max_) identified across all STD spectra. A positive STD signal was categorized as “strong”, “moderate” or “weak” where the intensity was >50%, >25% or <25% of I_max_, respectively [30].

### 3.5. 2D ^15^N–^1^H HSQC Spectra

Protein binding by compounds identified by SPR and STD screens was confirmed by recording ^15^N–^1^H HSQC spectra on uniformly ^15^N-labeled DnaGC (100 μM) in the presence of 3.3 mM compounds (from ^2^H_6_-DMSO stocks) with HSQC buffer (50 mM MES pH 6.0, 60 mM NaCl, 1 mM dithiothreitol) containing 3% ^2^H_2_O. The final volume of each sample was 150 μL. The recording time was 30 min for each ^15^N–^1^H HSQC experiment. A standard pulse sequence was used for data acquisition. Spectra were recorded at 298 K with a Bruker Avance 600 MHz NMR spectrometer equipped with cryoprobe and auto-sample changer. Compounds were regarded as hits if chemical shift perturbation was observed in the ^15^N–^1^H HSQC spectra. The spectra were processed with TOPSPIN 3.1 and analyzed using CCPN [43]. Weighted CSP values [44] were calculated as 

CSP = (Δ*δ_H_*^2^ + 0.2 Δ*δ_N_*^2^)^0.5^

Binding affinities were estimated by incremental titration of fragments into protein, with recording of a ^15^N–^1^H HSQC experiment at each concentration point. Compound solubilities were tested to determine the highest concentrations of ligands used in assays. Equilibrium dissociation constants from NMR titration data were derived using the “single site-specific binding with ligand depletion” model in GraphPad Prism v.6.0 (La Jolla, CA, USA).

### 3.6. ^19^F-NMR Spectroscopy

1D ^19^F-NMR spectra were recorded on a Bruker Avance III 400 MHz NMR spectrometer (Bruker, Karlsruhe, Germany) equipped with the two-channel BBO probe with *z*-gradient at 300 K. All ^19^F-NMR spectra were recorded with 256 scans for fragment and complex samples sequentially. Fragments dissolved in ^2^H_6_-DMSO were diluted in HSQC buffer to give final fragment and protein concentrations of 1 mM and 50 μM, respectively.

### 3.7. Molecular Docking

AutoDock Tools 1.5.6 [45] was used to prepare protein [46] and ligand structures for docking. The protonation state of the titratable groups in the protein were assigned at pH 7.0 using PROPKA 3.1 [47]. Polar hydrogen atoms and atom-based Gasteiger partial charges were added. Nonpolar hydrogen atoms were merged with the parent atom. The DnaGC structure was taken from the previously solved crystal structure (PDB ID: 1T3W) [13]. The protein was treated as a rigid body. The CSP docking calculations were performed using AutoDock Vina 1.1.2 [45]. The calculations utilized an exhaustiveness of 1024 with grid points separated by 1.0 Å and grid size large enough to include the SSB-Ct peptide binding site (16 × 16 × 14 Å). Ligand data were obtained from the *ZINC* database of commercially available compounds [38].

## 4. Conclusions

Fragment-based screening has successfully identified compounds targeting the DnaG primase and its SSB-Ct interaction. Compounds containing indole and 1*H*-tetrazole scaffolds were identified as first-generation hits. Based on CSP-guided molecular modeling studies, they are involved in formation of various electrostatic and hydrogen bond networks in the binding pockets, which makes them promising starting points for further optimization. Initial in silico fragment-to-lead optimization was carried out using the *ZINC* Database. *Para*-phenyl substituted tetrazoles were identified. The observation that compounds selected for binding to the C-terminal domain of DnaG primase also bind to other SSB-interacting proteins indicates that compounds may in future be derived that bind to similar binding pockets in multiple protein targets, which is a prerequisite for development of antibacterial compounds with a very low propensity for development of resistance.

## Supplementary Materials

The following are available online at http://www.mdpi.com/2079-6382/7/1/14/s1, Figure S1. Surface plasmon resonance (SPR) competition sensorgram for one of the SPR hits, and saturation transfer difference (STD)-NMR spectrum of SPR hit **D6**; Figure S2. ^15^N–^1^H HSQC spectra of DnaGC protein with and without fragment hits **1**–**3**; Figure S3. Residue-specific weighted chemical shift perturbations induced in ^15^N-DnaGC by binding to compounds **1**–**6**; Figure S4. Modeled orientation of fragment **1**; Figure S5. Comparison of chemical shift perturbation (CSP) induced by fragment **1** and compound **L1C6**; Figure S6. Modeled orientation of fragment **3**; Figure S7. Modeled orientation of *N*-acetyl-l-Phe (green carbon atoms) in the SSB-Ct binding pocket of DnaGC (gray carbon atoms). Figure S8. STD NMR spectrum of fragment **4** with four SSB-Ct binding partners other than DnaG primase.
