# Supplementary material for: Fragment-Based Discovery of Inhibitors of the Bacterial DnaG-SSB Interaction
Source: Antibiotics (Basel). 2018 Feb 22;7(1):14. doi: 10.3390/antibiotics7010014 (PMC5872125; doi:10.3390/antibiotics7010014)
Supplement: Supplementary file 1 [file antibiotics-07-00014-s001.pdf]

Supplementary Materials

Fragment-Based Discovery of Inhibitors of the Bacterial DnaG–SSB Interaction

Zorik Chilingaryan [1], Stephen J. Headey [2], Allen T. Y. Lo [1], Zhi-Qiang Xu [1], Gottfried Otting [3], Nicholas E. Dixon [1], Martin J. Scanlon [2] and Aaron J. Oakley [1,*]

[1] Molecular Horizons and School of Chemistry, University of Wollongong, and Illawarra Health and Medical Research Institute, Wollongong, NSW 2522, Australia; zorik@uow.edu.au (Z.C.); zhiqiang@uow.edu.au (Z.-Q.X); nickd@uow.edu.au (N.E.D.)

[2] Monash Institute of Pharmaceutical Science, Monash University, Parkville, VIC 3052, Australia; stephen.headey@monash.edu (S.J.H.); martin.scanlon@monash.edu (M.J.S.)

[3] Research School of Chemistry, Australian National University, Canberra, ACT 2601, Australia; gottfried.otting@anu.edu.au

* Correspondence: aarono@uow.edu.au; Tel.: +61-2-4221-4347

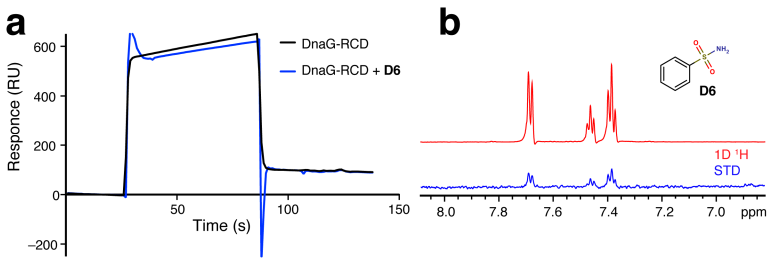

Figure S1. (**a**) SPR competition sensorgrams for one of the SPR hits, where the protein alone is in black and in blue when mixed with fragment **D6**. (**b**) STD spectrum of SPR hit D6 confirmed by STD-NMR. The red trace shows the 1D ^{1}H-NMR spectrum of ligand alone and in blue is the final STD spectrum.

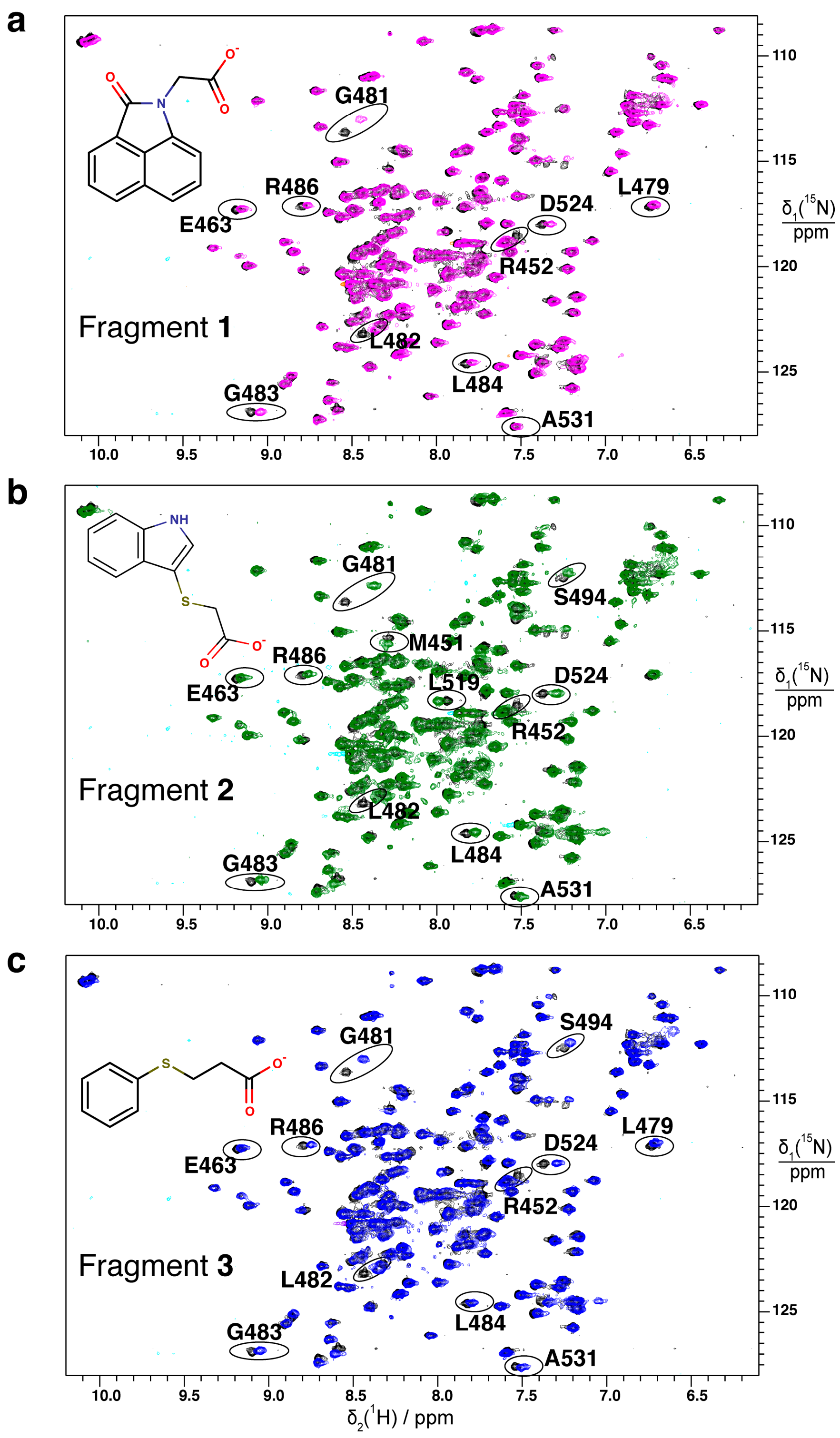

Figure S2. ^{15}N–^{1}H HSQC spectra of DnaGC protein with and without fragment hits **1**–**3**. (**a**) Fragment **1**; (**b**) fragment 2; (**c**) fragment 3. The spectrum of the DnaGC protein alone is shown in black, with the colored spectra recorded after addition of the fragments. Structures of the fragments and representative assignments of resonances that showed the highest weighted CSP (Figure S3a–c) are shown.

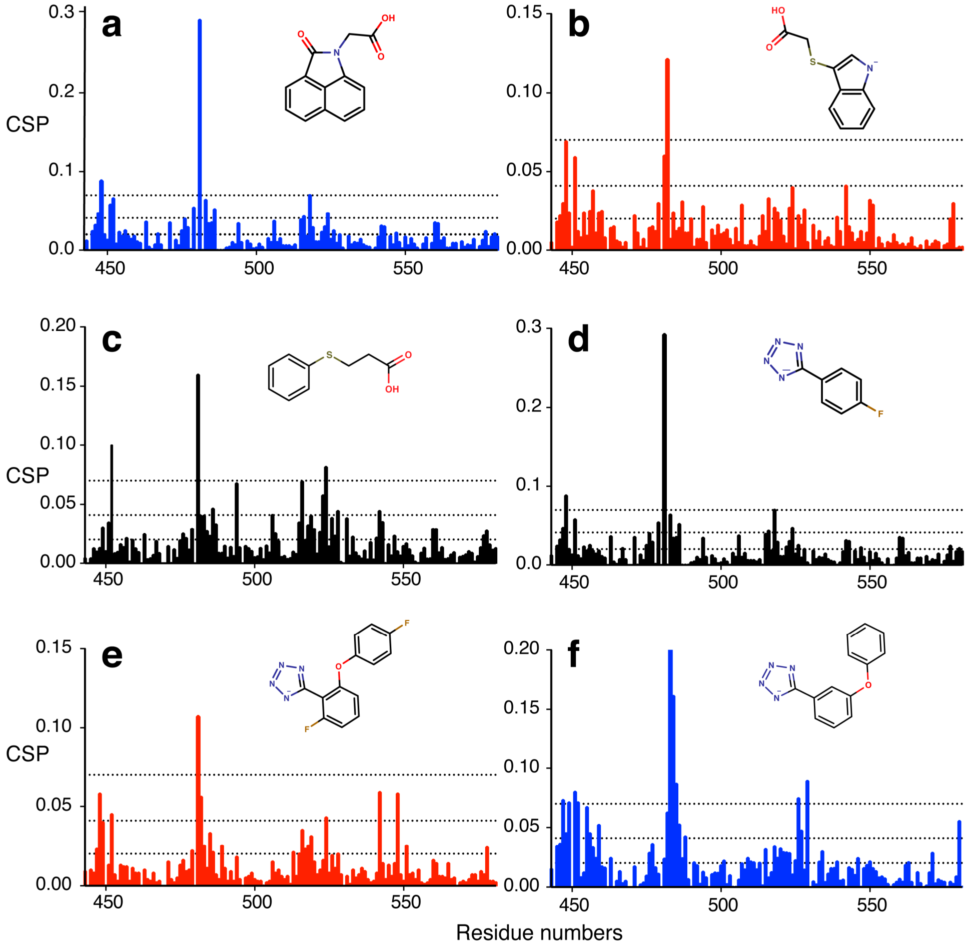

Figure S3. Residue-specific weighted chemical shift perturbations induced in ^{15}N-DnaGC by binding to fragments **1** (**a**), **2** (**b**), **3** (**c**), and **4** (**d**), and to compounds **5** (**e**) and **6** (**f**) monitored by ^{15}N–^{1}H HSQC NMR spectra.

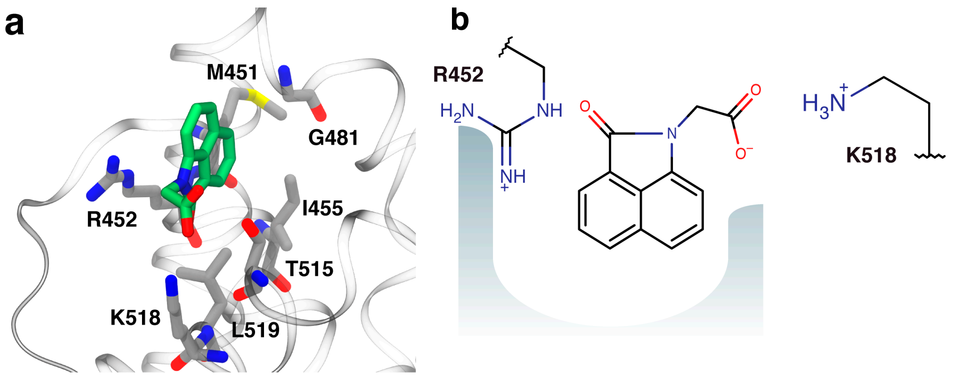

Figure S4. Modeled orientation of fragment **1**. (**a**) Predicted orientation of the hit (green carbon atoms) in the SSB-Ct binding pocket of DnaGC (gray carbon atoms). (**b**) Schematic representation of ionic and H-bond interactions between the protein and fragment **1**.

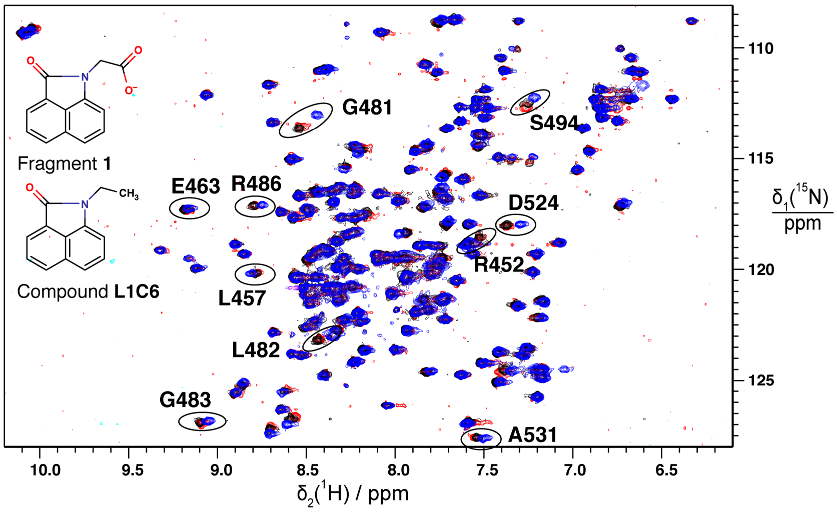

Figure S5. Comparison of CSP induced by fragment **1** (blue) and compound **L1C6** (red), with chemical structures of two fragments. The ^{15}N–^{1}H HSQC spectrum of the DnaG protein alone is shown in black.

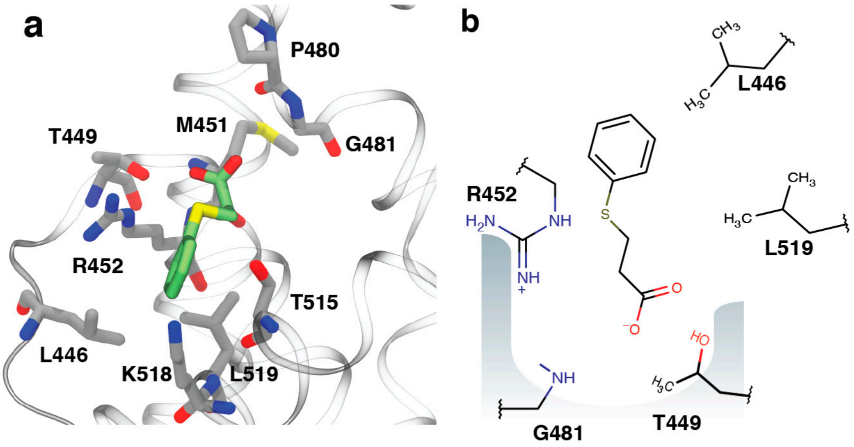

Figure S6. Modeled orientation of fragment **3**. (**a**) The docked orientation of fragment **3** (green carbon atoms) in the SSB-Ct binding pocket of DnaGC (gray carbon atoms). (**b**) A schematic representation of interactions between fragment **3** and its binding pocket.

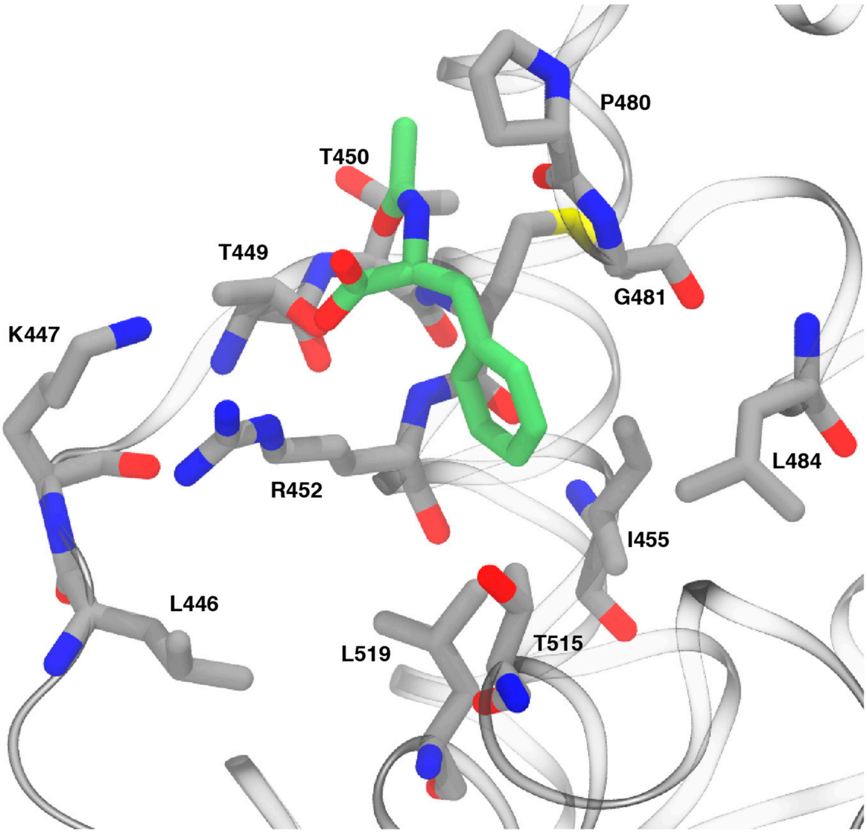

Figure S7. Modeled orientation of *N*-acetyl-L-Phe (green carbon atoms) in the SSB-Ct binding pocket of DnaGC (gray carbon atoms).

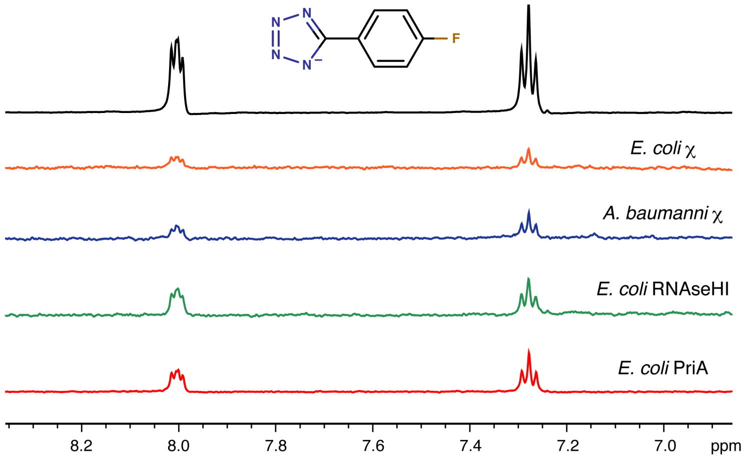

Figure S8. STD NMR spectrum of fragment **4** with four SSB-Ct binding partners other than DnaG primase. The top spectrum is the 1D ^{1}H-NMR spectrum of fragment **4** alone, and the other four spectra show STD spectra in the presence of each of the proteins, as indicated.
